# Impact of vascular architecture, oxygen saturation, and hematocrit on human cortical depth-dependent GE- and SE-BOLD fMRI signals: A simulation approach using realistic 3D vascular networks

**DOI:** 10.1162/imag_a_00573

**Published:** 2025-05-09

**Authors:** Mario Gilberto Báez-Yáñez, Jeroen C.W. Siero, Vanja Curcic, Matthias J.P. van Osch, Natalia Petridou

**Affiliations:** Translational Neuroimaging Group, Center for Image Sciences, University Medical Center Utrecht, Utrecht, The Netherlands; Spinoza Centre for Neuroimaging Amsterdam, Amsterdam, The Netherlands; C.J. Gorter MRI Center, Department of Radiology, Leiden University Medical Center, Leiden, The Netherlands

**Keywords:** 7 tesla, biophysical modeling, laminar BOLD fMRI, layer fMRI, Monte Carlo simulations, oxygen saturation levels, realistic cortical vascular network, vessel size dependent hematocrit

## Abstract

Recent advancements in MRI hardware, including ultra-high magnetic field scanners (≥7T) and MR data acquisition methods, have enhanced functional imaging techniques, allowing for the detailed study of brain function, particularly at the mesoscopic level of cortical organization. This has enabled the measurement of blood oxygenation level-dependent (BOLD) functional magnetic resonance imaging (fMRI) signal changes across cortical depth in the human brain, facilitating the study of neuronal activity at laminar level. In order to better understand the generation of cortical depth-dependent BOLD signals, biophysical modeling and computational simulations permit the characterization of the impact of vascular architecture, as well as the biophysical and hemodynamic effects at the mesoscopic level. In this study, we employed four realistic 3D vascular models that mimic the human cortical vascular architecture and simulated various vessel-dependent oxygen saturation and hematocrit states, aiming to characterize the intravascular and extravascular contributions to gradient-echo (GE) and spin-echo (SE) BOLD signal changes across human cortical depth at 7T. We found that differences in the local vascular architecture between the four models, away from the pial surface, do not significantly influence the shape and amplitude of BOLD profiles. This implies that signal profiles within a cortical region of a given angioarchitecture can be averaged within a given layer without introducing substantial errors in the results. The findings futher reveal that in deeper laminae, relative relaxation rates for both GE and SE decrease linearly with increasing oxygen saturation levels, with GE showing a stronger effect. In contrast, the top lamina shows a non-linear behavior due to large vessel contributions, particularly venous, with GE displaying higher relaxation rates (4–8 times larger dependent on oxygen saturation levels) than SE. Relative BOLD signal changes also follow linear trends in deeper layers, with GE peaking at ~8% and SE at ~4%, reflecting the higher microvascular specificity of SE. However, SE does not fully eliminate large vessel contributions at the pial surface, where diffusion effects and vessel architecture play a role. Hematocrit levels linearly change the BOLD signal amplitude and significantly influence laminar contributions across cortical depth and imaging techniques. While GE signals are dominated by extravascular effects, SE retains notable intravascular venous contributions at high oxygen saturation levels, which is particularly relevant in experiments involving controlled vascular oxygenation, that is, gas challenges. These results underscore how vascular features, hematocrit, and biophysical interactions shape cortical depth-dependent BOLD signals and their specificity in ultra-high field imaging.

## 1 Introduction

Functional magnetic resonance imaging (fMRI) has revolutionized our understanding of human brain function by allowing noninvasive mapping of brain activity. One of the most commonly used fMRI techniques relies on the blood oxygenation level-dependent (BOLD) signal, which is influenced by local changes in cerebral blood flow (CBF), cerebral blood volume (CBV), and rate of oxygen compsuption (CMRO_2_) in response to neuronal activity (Bandettini et al., 1994,^1997^;Ogawa et al., 1993).

Due to recent advances in MRI hardware, such as ultra-high magnetic field scanners (≥7 tesla), dedicated coils, imaging gradients, and MR data acquisition strategies, BOLD imaging techniques now enable the study of brain function at a high level of detail, specifically at the level of the mesoscopic organization of the human cortex (Harel et al., 2006;Koopmans et al., 2010,^2011^;Ress et al., 2007). Consequently, it is now possible to measure BOLD fMRI signal changes as a function of cortical depth—that is, laminar BOLD fMRI—in the human cortex and study neuronal activity across different cortical layers (De Martino et al., 2013;Dumoulin et al., 2018;Fracasso et al., 2018;Huber et al., 2017;Kashyap et al., 2018;Markuerkiaga et al., 2021;Nasr et al., 2016;Olman et al., 2012;Siero et al., 2011). These breakthroughs underscore the potential of high-resolution fMRI as a valuable tool for investigating the fundamental processing within cortical micro-circuits and their intricate interactions (Gau et al., 2020;Kok et al., 2016).

While laminar BOLD fMRI holds great promise for advancing our understanding of cortical function, several challenges remain. Acquiring high-resolution BOLD fMRI data requires careful optimization of imaging parameters to balance spatial resolution, sampling rate, signal-to-noise ratio, and coverage (Norris & Polimeni, 2019;Polimeni & Uludağ, 2018). Laminar BOLD fMRI analysis techniques are still being refined, and validation against invasive techniques is necessary to confirm their accuracy (Choi et al., 2022;Lu et al., 2004;Poplawsky et al., 2019b;Silva & Koretsky, 2002;Yu et al., 2014;Zaldivar et al., 2018). Furthermore, the BOLD signal is only an indirect measurement of neuronal functioning. It is a mixture of effects related to hemodynamic changes induced by neurovascular coupling, the vascular architecture within the sampled volume, and the biophysical interaction of (de)-oxygenated blood and tissue. Because BOLD fMRI measures neuronal activity through hemodynamics, its ultimate resolution relies on the spatial extent of neuronal-evoked hemodynamic changes, along with how these changes evolve over time (Petridou & Siero, 2019;Puckett et al., 2016;Siero et al., 2013).

Numerous laminar fMRI studies, with spatial resolutions reaching ≤1 mm in all directions, have examined the spatial and temporal characteristics of the recorded hemodynamic signals across cortical depth (Dumoulin, 2017;Kotlarz et al., 2023;Polimeni et al., 2010;Stephan et al., 2019). Gradient-echo (GE) BOLD is the most commonly used technique to measure brain activation across cortical depth due to its straightforward implementation. Nevertheless, it is well documented that GE-BOLD is sensitive to both large vessel and microvascular signal contributions, which impact the specificity to the underlying neuronal activity, especially toward the pial surface (Bause et al., 2020;Han et al., 2022).

Spin-echo (SE) BOLD, on the other hand, increases specificity toward the microvasculature (van Kerkoerle et al., 2017;Zhao et al., 2006) and is, therefore, considered to be more specific to the location of neuronal activity (Norris, 2012;Pflugfelder et al., 2011;Uludağ et al., 2009). This has been demonstrated using computational simulations based on mono-sized, randomly oriented cylinder models (Boxerman et al., 1995;Kiselev, 2001;Kiselev & Posse, 1999;Weisskoff et al., 1994). However, SE-BOLD fMRI measurements have shown that large vessel contributions may not be completely removed (Goa et al., 2014;Pfaffenrot et al., 2021;Pflugfelder et al., 2011). Large vessels may still influence the detected BOLD signal changes, especially near the pial surface (Bause et al., 2020), due to several factors such as the impact of the echo planar imaging (EPI) readout (Goense & Logothetis, 2006;van Horen et al., 2023), dependence on the echo time (TE) selection (Havlicek et al., 2017;Markuerkiaga et al., 2016), intrinsic pulse sequence characteristics, for example, pial vessel contributions in SE-like S2 nbSSFP signals (Goa et al., 2014), or the different diffusion regimes between the gray matter (GM) and the cerebrospinal fluid (CSF) (Kiselev & Posse, 1999;Yablonskiy & Sukstanskii, 2010).

To gain a better understanding of the impact of human cortical vascular architecture and intrinsic hemodynamics on the generation of cortical depth-dependent BOLD signals, it is necessary to investigate and characterize both the intravascular and extravascular contribution to GE-BOLD and SE-BOLD signal formation across cortical depth using computational simulations based on realistic 3D vascular networks.

The aim of this study is to investigate the influence of human cortical vascular architecture (including topology and CBV distribution) and intrinsic hemodynamics, considering various oxygen saturation and hematocrit states, on the characterization of MR relaxation rates and BOLD signal changes across cortical depth. To achieve this, we utilized four realistic 3D vascular models of mice obtained using two-photon microscopy imaging techniques (Blinder et al., 2013). These models were then adapted by modifying the artery-to-vein ratio to mimic features of the human cortical vasculature (Duvernoy et al., 1981;Reina-De La Torre et al., 1998). Simulations were performed to evaluate different vessel-dependent oxygen saturation and hematocrit states, as well as the biophysical interactions between (de)-oxygenated blood and tissue, in order to characterize the intravascular and extravascular contributions to GE-BOLD and SE-BOLD signal changes across cortical depth at 7 tesla (7T).

## 2 Material and Methods

### 2.1 Simulation of 3D human cortical vascular models using two-photon microscopy data from mice

**Fig. 1.**
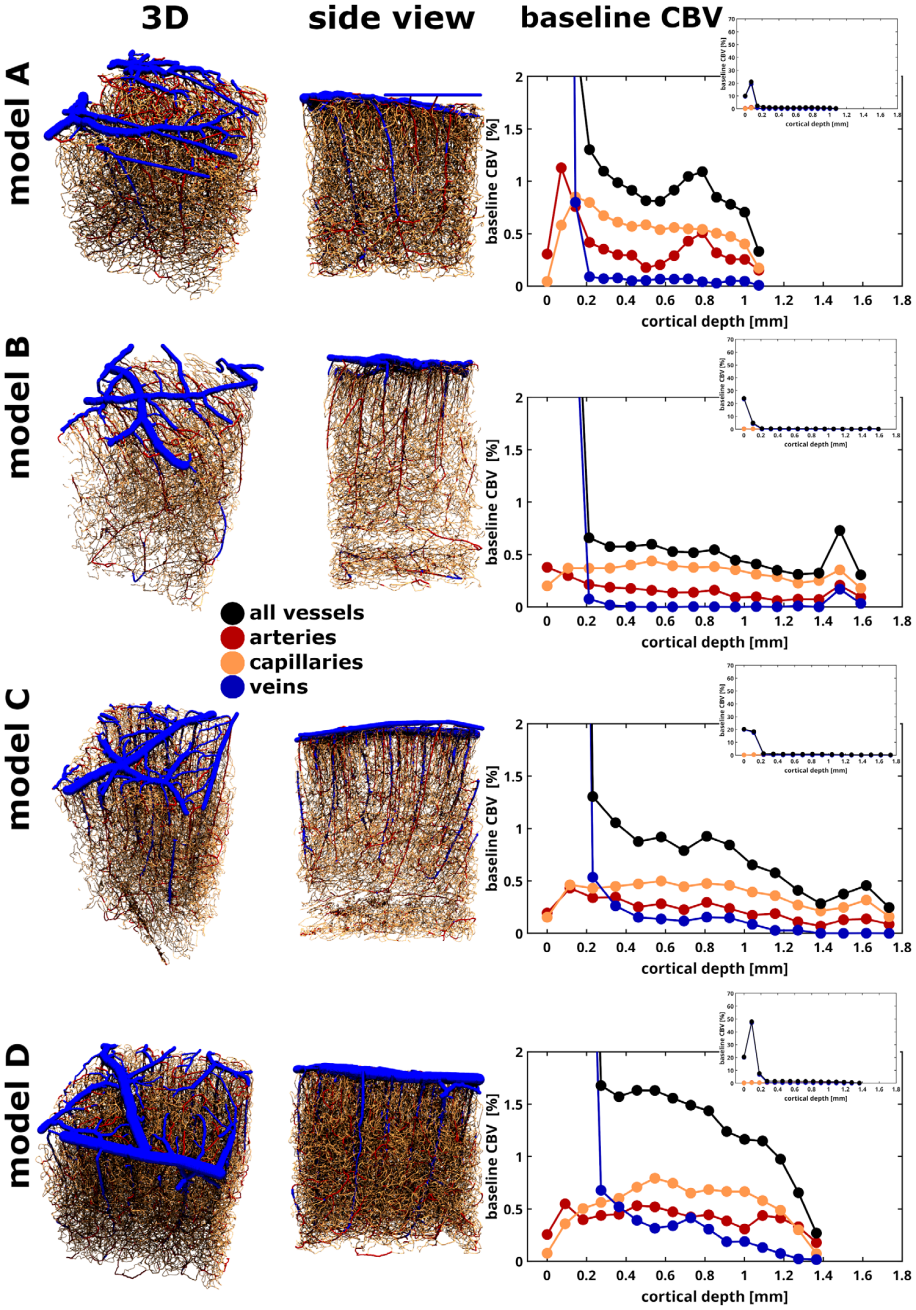
Four realistic 3D vascular models (A–D models) were created using two-photon microscopy imaging techniques (Blinder et al., 2013). These models include three vessel types: macrovessels, represented by red arteries and blue veins, and microvessels, consisting of capillaries, small arterioles, and venules, depicted in beige. To reflect human brain characteristics, the macrovessels were labeled with an approximate 3:1 artery-to-vein ratio. The pial surface is positioned at 0 [mm] for all models. On the right side of each model, baseline CBV features (range: 0–2% blood volume) are shown for both macrovessels (indicated by red and blue solid lines with dots) and microvessels (shown with beige solid lines with dots), and the sum from all vessels is represented by black solid lines with dots. The upper-right panels show a zoomed-out view of the total CBV.

We used four samples of 3D vascular mouse models acquired with two-photon microscopy imaging techniques from the barrel cortex, for example, primary sensory cortex, as obtained byBlinder et al. (2013)(Fig. 1). The vectorized vascular data are openly available on the Kleinfeld Lab website (https://neurophysics.ucsd.edu/journal_articles.php#year_2011). Using custom code in MATLAB (MathWorks, v.2022a), we reconstructed the vasculature using the spatial coordinates of the vessel segments and their associated diameter and length information. The vascular data provided byBlinder et al. (2013)were stored in a vectorial structure, where spatial positions comprised an m-by-3 vector, with m representing the number of vessel segments in the vascular model, and two m-by-1 column vectors representing the diameter and length of each vessel segment, respectively. Capillaries were effectively represented by their length and diameter due to their relatively uniform structure. However, arteries and veins, with their intricate shapes, required subdivision into multiple segments to accurately capture their branching patterns, varying diameters, and curvature. More details on the vascular data (e.g., vessel radius, length, curvature, or preferential orientation with respect to the cortical surface) and the connectivity of the vessel segments can be found inBlinder et al. (2013)andBáez-Yáñez et al. (2017).

In this work, vessels smaller than 6 micrometers in radius were labeled as capillaries (Schmid et al., 2019), and larger vessels were designated as macro-vessels. The labeling of the vessels into arteries or veins was done using the radius values of the large vessels near the pial surface. Vessels with radius ranging from 11 to 36 µm were designated as veins, while those with radius from 6 to 11 µm were labeled as arteries (Duvernoy et al., 1981;Reina-De La Torre et al., 1998). Once categorized as either an artery or a vein, the penetrating vessel segments were labeled as artery or vein depending on the nearest larger vessel segment label, similar to following the vessel path, across cortical depth. To replicate human brain characteristics, the macro-vessels were divided into a 3:1 artery-to-vein ratio, reflecting the ~3:1 ratio observed in humans (Duvernoy et al., 1981;Reina-De La Torre et al., 1998) compared to the approximately 1:3 ratio measured in mice (Blinder et al., 2013;Stamenkovic et al., 2024;Uludağ & Blinder, 2018).

Thus, the resulting 3D human vascular models included arteries and veins (referred to as macrovessels from here onward) along with small arterioles, capillaries, and venules (referred to as microvessels). The volume size of the vascular models was about 1 to 1.75 mm in cortical thickness, that is, along the z-direction. The xy-plane was approximately 1 mm^2^for all models.

We further defined, arbitrarily, 16 laminae (slabs of tissue covering the respective vessels which result in averaged sample points) evenly distributed between the pial surface (cortical depth = 0 mm) and the white matter/gray matter (WM/GM) boundary (bottom of each model, seeFig. 1). The laminar thickness was variable and dependent on the cortical thickness of each vascular model. It is important to note that we chose 16 equally spaced sample points along the cortical depth instead of the well-known cortical layer division in humans to obtain more sample points for computations. The baseline CBV of each lamina was computed as the sum of the volume of all its vessels. The volume of a single vessel was calculated from its diameter and length, assuming a cylindrical shape (seeFig. 1).

### 2.2 Implementation of vessel-dependent oxygen saturation levels

We simulated 15 different oxygen saturation (SO _2_ ) levels per vascular compartment, which were maintained constant over time, that is, steady-state oxygen saturation levels were assumed ( Vovenko E., 1999 ). The oxygen saturation values used in the microvessels depended on the SO _2_ imposed on the veins, as follows:

- SO_2_in arteries (SO_2_art) = 95%;
- SO_2_in microvessels = SO_2_art – ((SO_2_art – SO_2_vein) / 2);
- SO_2_in veins (SO_2_vein) = [50%, 53%, 56%, 59%, 62%, 65%, 68%, 71%, 74%, 77%, 80, 83%, 86%, 89%, 92%].

The 95% SO_2_value for the arterial compartment was selected based on the assumption that it represents the minimum expected value to be measured in this compartment. Increments in the steady-state arterial SO_2_value, within the range of 96% to 100%, would likely reduce the arterial contribution to the MR signal formation.

Furthermore, we extended the simulated oxygen saturation range beyond physiologically plausible values—from 60% SO_2_in veins during the resting state to 80% during the active state—to gain a broader understanding of the BOLD signal formation process under experimental conditions, such as during induced gas-challenges (hyperoxia) experiments (Báez-Yáñez et al., 2021;Schellekens et al., 2023).

### 2.3 Implementation of vessel size-dependent hematocrit level

The formation of the BOLD signal is fundamentally influenced by systemic hemoglobin concentration, that is, hematocrit (Hct), and variations in hematocrit between the different vascular compartments may induce differences in cortical depth-dependent BOLD signal changes ( Gould & Linninger, 2015 ). In this study, we simulated a vessel size-dependent hematocrit level using experimental values obtained by Gould and Linninger (2015) as follows:

- arteries: 0.90 * Hct;
- microvessels: 0.70 * Hct;
- veins: 1.20 * Hct,

The weighting value (1.20) for the veins was chosen to represent a plausible extreme scenario in which red blood cells accumulate, leading to a stalling effect in this vascular compartment, particularly in the venules. Since the BOLD signal is highly sensitive to venous contributions, and our capillary bed does not clearly distinguish between arterioles, small capillaries, and venules, we opted to simulate this effect in the larger venous compartment as a proxy for its impact on the BOLD signal (Gould & Linninger, 2015). Reduced weighting values in the veins would likely only decrease their contribution to the MR signal.

From here onward, we used the systemic hematocrit definition to set the values in the simulations, ensuring that the Hct value always corresponded to the vessel size characteristics as defined previously.

We conducted simulations for two distinct scenarios. In the first, we examined the effects of a fixed hematocrit level of 45%. In the second, we investigated the impact of varying Hct levels on the BOLD signal amplitude by simulating a range of Hct values from 35% to 55%. Further details on these simulations are provided inSection 2.6.

### 2.4 Simulation of the MR signal accounting for both intravascular and extravascular signal contributions

In this study, BOLD fMRI signals consisted of both extravascular and intravascular components influenced by the vascular architecture and baseline CBV (seeFig. 1), SO_2_(seeSection 2.2), and hematocrit (seeSection 2.3).

The total MR signal,S(t), was calculated by summing the extravascular signal contribution,$S_{EV}$, with both arterial,$S_{aIV}$, and venous,$S_{vIV}$, intravascular signal contributions, each weighted by their respective baseline blood volume:

$$S\left(t\right)=\;S_{EV}\left(t\right)+S_{aIV}\left(t\right)+S_{vIV}\left(t\right)\;$$ (1)

The simulations presented here were computed for GE and SE pulse sequences at 7T, assuming an instantaneous readout. Additionally, we assumed the angular orientation of the normal vector arising from the pial surface of the 3D vascular model to be parallel to the main magnetic field$B_{0}$(Báez-Yáñez et al., 2023;Fracasso et al., 2018;Gagnon et al., 2015;Viessmann et al., 2019).

#### 2.4.1 Arterial and venous intravascular signal contributions

The intravascular signal contribution for each vascular compartment can be approximated as follows:

$$\begin{array}{l} S_{aIV}\left(t\right)=\left(bCBV_{a}\right)\;\cdot \;\left(e^{-R2_{\;dHb}^{*}\cdot \;t}\right)\;\;\;\;\;for\;GE;\;\\ and\;\;\;\;\;S_{aIV}\left(t\right)=\left(bCBV_{a}\right)\;\cdot \;\left(e^{-R2_{dHb}\cdot \;t}\right)\;\;\;\;\;for\;SE\; \end{array}$$ (2)

And

$$\begin{array}{l} S_{vIV}\left(t\right)=\left(bCBV_{v}\right)\;\cdot \;\left(e^{-R2_{\;dHb}^{*}\cdot \;t}\right)\;\;\;\;\;for\;GE;\;\\ and\;\;\;\;\;\;S_{vIV}\left(t\right)=\left(bCBV_{v}\right)\;\cdot \;\left(e^{-R2_{dHb}\cdot \;t}\right)\;\;\;\;\;for\;SE\; \end{array}$$ (3)

where$bCBV_{a}$for arteries and$bCBV_{v}$for veins represent the baseline blood volume fractions (seeFig. 1);$R2_{\;dHb}^{*}$and$R2_{dHb}$represent the sum of the intrinsic relaxation rate of blood,$R2_{\;0,in}^{*}$or$R2_{0,in}$, and the effect induced by the oxygen saturation level,$R2_{SO2}$for GE and SE, respectively, as follows:

$$\begin{array}{l} R2_{\;dHb}^{*}=\;R2_{\;0,in}^{*}+R2_{SO2}\;\;\;\;\;for\;GE;\;and\;\;\;\;\\ R2_{dHb}=\;R2_{0,in}+R2_{SO2}\;\;\;\;\;for\;SE\; \end{array}$$ (4)

We assumed that the intravascular signal contribution to the MR signal is non-zero for both the arterial and venous compartments. This assumption is based on the observation that at high magnetic fields, the MR signal contributions from both arterial and venous compartments, particurlarly in the veins, are not negligible at higher (≥80%) SO_2_levels (Boxerman et al., 1995;Uludağ et al., 2009;Weisskoff et al., 1994).

On the other hand, we assumed the intravascular signal contribution of the microvascular compartment to be null, given that the$R2_{\;dHb}^{*}$and$R2_{dHb}$of the capillaries has not been well characterized, and the intravascular contribution of capillaries is not expected to be as significant as that of larger vessels.

Therefore, we implemented the intravascular arterial and venous signal contribution for GE using$1/R2_{\;0,in}^{*}=$$T2_{\;0,in}^{*}$(≈10.00 ms) (Siero et al., 2013), and$R2_{SO2}$component dependent on SO_2_using the quadratic relation as defined byUludağ et al. (2009)(seeTables 1and2), weighted by the corresponding blood volume fraction (seeFig. 1). Similarly, the intravascular arterial and venous signal contributions for SE as$1/R2_{0,in}=T2_{0,in}$(≈53.82 ms) (Siero et al., 2013), and$R2_{SO2}$component dependent on SO_2_^39^(Uludağ et al., 2009) (seeTables 1and3).

**Table 1.** Biophysical and pulse sequence parameters used to compute a BOLD signal response.

**Biophysical and MR pulse sequence parameters**	
Diffusion coefficient (extravascular tissue)	1 µm ^2^ /ms; isotropic motion
Static magnetic field strength	7T
Systemic hematocrit (Hct) (used in all Figures except Fig. 7 )	45%
Systemic hematocrit (HcT) (used in Fig. 7 )	35% to 55% with increments of 2.5%
Echo-time (TE)	gradient-echo = 27 ms spin-echo = 50 ms
Time-step	0.025 ms
Number of spins	20 Monte-Carlo repetitions with 5 × 10 ^7^ spins for each repetition
GE: T2 _0_ * cortical grey matter at 7T SE: T2 _0_ cortical grey matter at 7T	28.57 ms 48.30 ms
GE: intravascular arterial T2* _dHb_ at 7T	9.87 ms @ SO _2_ = 95%
SE: intravascular arterial T2 _dHb_ at 7T	49.67 ms @ SO _2_ = 95%
GE: intravascular venous T2* _dHb_ at 7T	see Table 2 .
SE: intravascular venous T2 _dHb_ at 7T	see Table 3 .

**Table 2.** GE: intravascular venous T2*_dHb_at 7T.

SO _2_ [%]	50	53	56	59	62	65	68	71	74	77	80	83	86	89	90
T2* _dHb_ [ms]	4.41	4.72	5.05	5.40	5.78	6.17	6.59	7.01	7.45	7.89	8.31	8.72	9.09	9.42	9.68

**Table 3.** SE: intravascular venous T2_dHb_at 7T.

SO _2_ [%]	50	53	56	59	62	65	68	71	74	77	80	83	86	89	90
T2 _dHb_ [ms]	5.75	6.42	7.20	8.13	9.23	10.56	12.17	14.12	16.51	19.44	23.03	27.38	32.52	38.32	44.33

#### 2.4.2 Extravascular signal contribution

**Fig. 2.**
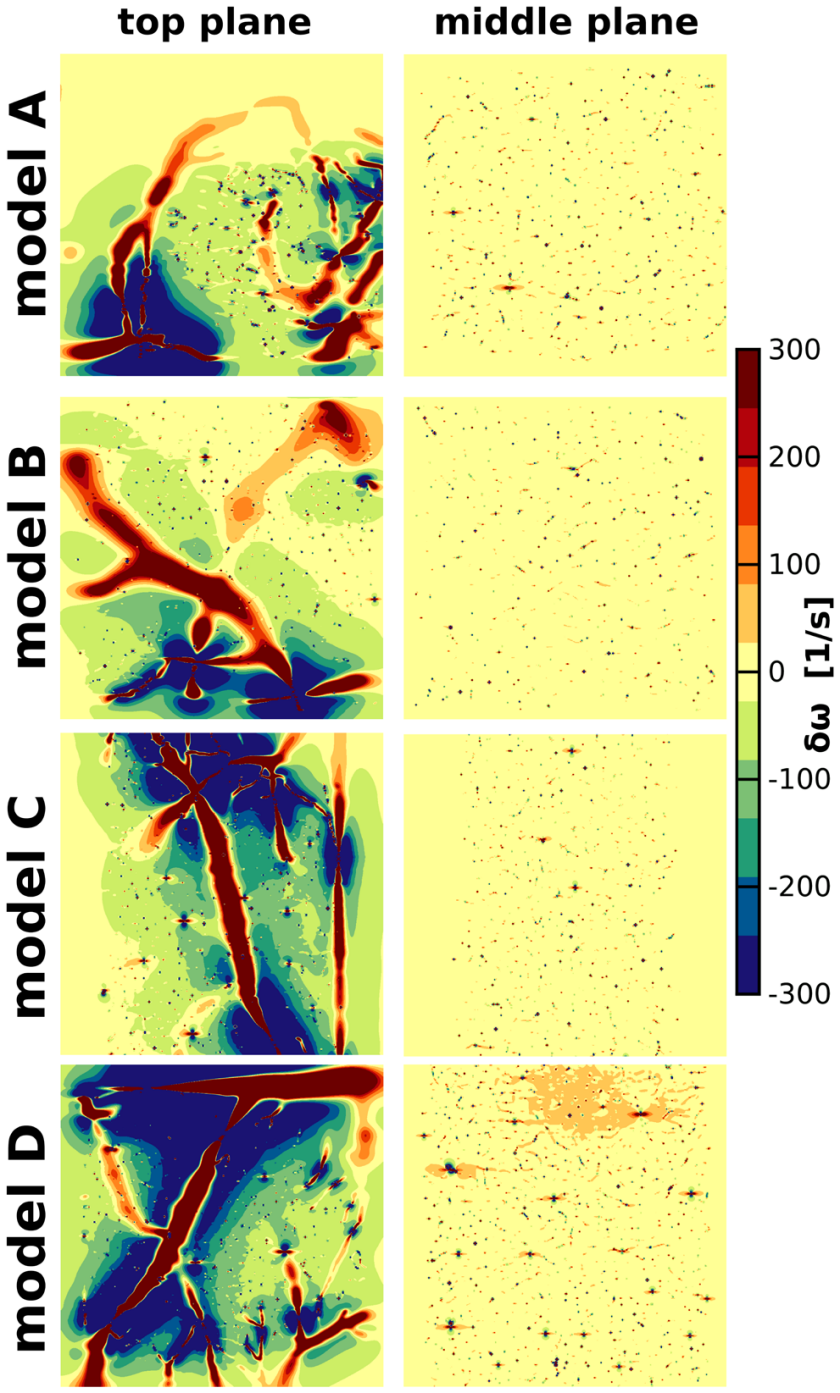
Local frequency field distortions produced by the realistic vascular models for an exemplary oxygen saturation level (SO_2_vein = 78%) and hematocrit (Hct = 45%) are shown. The oxygen saturation value was arbitrarily selected for illustration purposes. The frequency field maps are depicted for two different locations inside the simulated voxel: the top plane, situated approximately 20 μm deep from the cortical pial surface, and the middle plane, positioned around 500 μm deep relative to the pial surface. The main magnetic field was simulated to be orthogonal to the cortical surface and thus to the depicted planes.

The extravascular signal contribution$S_{EV}\left(t\right)$was computed by modeling the interaction of moving spins within the local inhomogeneous magnetic field induced by the varying SO_2_levels (Boxerman et al., 1995;Weisskoff et al., 1994) (Section 2.2). We computed local frequency shifts created by the SO_2_levels of both the macro- and microvascular compartments (Fig. 2).

The local frequency shift caused by a vessel segment was modeled as the dipolar induced-response of a finite cylinder, presuming negligible effects on the cylinder extremities (Báez-Yáñez et al., 2023;Kiselev & Posse, 1999). The local frequency shiftδω(r)in [1/s] for each vessel segment was computed using:

δω(r)=12 · γ2π · B0 · Δχ · (R2r2) · cos(2θ) · sin2ψ  (5)

where γ is the hydrogen gyromagnetic ratio = 267.5 × 10^6^[rad∙(s ·  T)^-1^],$B_{0}$is the main magnetic field (7 [T]),$\Delta \chi =4\pi \;\cdot \;0.276\;ppm\;\cdot \;HcT\left(R\right)\;\cdot \;\left(1\;\;-\;SO_{2}\right)$[-] is the susceptibility difference produced by the$SO_{2}$in the vessel (Kiselev, 2001) and the vessel size-dependent hematocritHct(R),Ris the vessel radius in [µm],ris the Euclidean distance from the center line of the vessel to a particular spatial position in the simulation volume in [µm],θis the angle between the vessel and the spatial position in [rad], andψis the angle between the orientation of the vessel and the main magnetic field in [rad].

The dephasing experienced by a bulk of diffusing spins$N_{spins}$was simulated using a Monte Carlo approach with 20 repetitions and 5 × 10^7^spins for each repetition. An ensemble of spins senses the extravascular local frequency shifts with a diffusion coefficient of D = 1.0 [µm^2^∙ms^-1^], while assuming isotropic diffusion (Kiselev & Novikov, 2018). The calculation of the spin dephasing was obtained through,

$$\varphi \left(t\right)=\int_{0}^{t}\delta \omega \left(x\left(t\right)\right)\;dt$$ (6)

whereφ(t)is the phase acquired during the simulation timetandδω(x(t)) is the local frequency shift at spin positionxat each time-stept. The dephasing experienced for each spin was stored across all simulation time-steps (time step = 0.025 ms). For SE sequences, the acquired dephasing after TE/2 was multiplied by -1 (change in polarity) simulating the effect of the 180-degree refocusing radiofrequency pulse. UsingEquation (7)we can obtain the extravascular MR signal contribution.

$$\begin{array}{cc} S_{EV}\left(t\right)\;\;=\;\;\left(1\;\;-\;\;\left(bCBV_{a}\;\;+\;\;bCBV_{v}\right)\right)\;\cdot \;[R2^{\prime }\;\cdot \;e^{-R2_{\;0}^{*}\cdot \;t}] & for\;GE;\\ S_{EV}\left(t\right)\;\;=\;\;\left(1\;\;-\;\;\left(bCBV_{a}\;\;+\;\;bCBV_{v}\right)\right)\;\cdot \;[R2^{\prime }\;\cdot \;e^{-R2_{0}\cdot \;t}] & for\;SE \end{array}$$ (7)

whereR2′is the relaxation rate induced by the interaction of the diffusing spins in a local inhomogeneous frequency field and dependent on pulse sequence, and is defined as,

$$R2^{\prime }=\left(\frac{1}{N_{spins}}\sum^{N_{spins}}e^{-i\varphi \left(t\right)}\right)\;$$ (8)

and$R2_{\;0}^{*}$= 1/T2_0_* and$R2_{0}$= 1/T2_0_are the effective relaxation rate of cortical tissue for GE and SE, respectively. We used the effective tissue T2_0_* (≈28 ms) relaxation time for GE and T2_0_(≈50 ms) relaxation time for SE according to the nonlinear relationship given byKhajehim and Nasiraei Moghaddam (2017)for gray matter at 7T (seeTable 1).

To confine the ensemble of spins within the simulation space, voxel boundary conditions were set to infinite space. Spins exiting the voxel re-entered the voxel volume on the opposite side, preserving their dephasing history. However, spins reaching the pial surface and WM/GM boundary were considered invalid iterations and were recomputed. Additionally, spin exchange between extra/intra-vascular compartments was prohibited, establishing an impermeable vascular network (Uludağ et al., 2009).

### 2.5 Relaxation rates and BOLD signal changes

Given that the behavior ofS(t)in (Eq. 1) presents oscillations due to its multi-exponential nature, we simply approximate the global relaxation rate$R2^{*}$andR2by fitting a polynomial of degree one, that is, a linear fit, on the natural logarithm ofS(t), that is,$R2^{\left(*\right)}=\frac{\ln\left(\text{S}\left(t\right)\right)}{t}$, for GE and SE, respectively.

In this manuscript, we present our results using two metrics, (1) the relative relaxation rate$\Delta R2^{\left(*\right)}$in [%], relative to the intrinsic relaxation rate of tissue (i.e., in the absence of any local susceptibility differences),

$$\Delta R2^{\left(*\right)}=\;\left(\frac{R2_{\;\;\;\lambda SO2}^{\left(*\right)}}{R2_{\;\;\;tissue}^{\left(*\right)}}-1\right)\times 100\;$$ (9)

where$R2_{\;\;\;\lambda SO2}^{\left(*\right)}$represents the varying oxygen saturation states as described inSection 2.2; and (2) the relative BOLD signal change,ΔBOLDin [%], which was defined as the relative change using the value of the exponential decay of the tissue at the TE as the baseline condition, that is, ($S{\left(TE\right)}_{tissue}=\text{exp}(-R2_{\;\;\;tissue}^{\left(*\right)}\cdot TE)$), for GE and SE respectively, as follows,

$$\Delta BOLD=\;\left(\frac{S{\left(TE\right)}_{\lambda SO2}}{S{\left(TE\right)}_{tissue}}-1\right)\times 100\;$$ (10)

where$S{\left(TE\right)}_{\lambda SO2}$represents the varying oxygen saturation states as described inSection 2.2.

### 2.6 Simulation experiments

We conducted four different simulation experiments (E.1–E.4):

E.1) In this experiment, we aimed to investigate the dependence of the relative change (in %) of$\Delta R2^{*}$andΔR2relaxation rates on oxygen saturation for GE (TE = 27 ms) and SE (TE = 50 ms). To achieve this, we performed Monte Carlo simulations (20 iterations) across the various SO_2_states (Section 2.2), assuming a systemic Hct of 45% (arteries: 0.9 * Hct, microvessels: 0.70 * Hct, veins: 1.20 * Hct).

We averaged the 16 arbitrarily defined laminae (sample points) across the cortical depth into four categories. Laminae 1–4: top (comprising the pial surface); laminae 5–8: top middle; laminae 9–12: middle bottom; and laminae 13–16: bottom (comprising the GM/WM boundary). Additionally, we calculated the proportion (ratio) of the relative change of$\Delta R2^{*}$andΔR2, that is,$\Delta R2^{*}/\Delta R2$as a proxy of microvascular specificity—given that SE is more specific to microvessel contributions (Siero et al., 2013).

E.2) In this experiment, we performed Monte Carlo simulations (20 iterations) to characterize the relative ΔBOLD signal changes and relative relaxation rates across cortical depth (using the previously defined laminae in E.1) for GE (TE = 27 ms) and SE (TE = 50 ms) for various SO_2_states (Section 2.2). We set the systemic hematocrit to 45% (arteries: 0.9 * Hct, microvessels: 0.70 * Hct, veins: 1.20 * Hct).

E.3) In this experiment, we characterized the extravascular and intravascular signal contributions (in %) across cortical depth (using the previously defined laminae in E.1) for GE (TE = 27 ms) and SE (TE = 50 ms). The signal contributions were obtained using Monte Carlo simulations with 20 iterations across various SO_2_levels and a systemic hematocrit of 45% (arteries: 0.9 * Hct, microvessels: 0.70 * Hct, veins: 1.20 * Hct).

E.4) In this experiment, we aimed to quantify the impact of different hematocrit values across cortical depth (using the previously defined laminae in E.1). For this, we conducted Monte Carlo simulations (20 iterations) across various SO_2_states (Section 2.2) for GE (TE = 27 ms) and SE (TE = 50 ms). We assumed systemic hematocrit values ranging from 35% to 55% (arteries: 0.9 * Hct, microvessels: 0.70 * Hct, veins: 1.20 * Hct).

The computational pipeline was implemented using custom code in MATLAB (MathWorks, v.2022a) and JULIA (v1.10.5). The biophysical parameters used to compute the relaxation rates and the respective BOLD signal changes are summarized inTable 1.

### 2.7 Validation of the computational pipeline

To validate the computational framework, we reproduced the results reported inBoxerman et al. (1995)andKiselev and Posse (1999). Using mono-sized randomly oriented cylinders, we computed ΔR2’ effects for GE and SE at 1.5T with identical simulation parameters used byKiselev and Posse (1999). This is shown inSupplementary Figure S1. Additionally, we calculated GE BOLD and SE BOLD signal changes at 7T to gain insights into the BOLD contrast using this simplified model, as detailed inSupplementary Figure S2.

## 3 Results

Figure 1illustrates the four realistic vascular models (A–D model) utilized in this study, including arteries, microvessels (capillaries and small arterioles/venules), and veins. Although the models were obtained from different mice within the same cortical region (barrel cortex, e.g., primary sensory cortex), the capillary beds in all four models demonstrate similar (i.e., similar order of magnitude) characteristics in vessel radius, baseline CBV, and vessel density across cortical depth (seeBáez-Yáñez et al., 2017;Blinder et al. 2013), with somewhat comparable topologies. However, the macrovascular architecture varies among the models, leading to distinct differences in large vessel organization. It is worth noting that the first 250 µm of cortical depth comprises a large blood volume fraction due to the large pial vessels. After this, the baseline CBV values show a decrease across cortical depth (see black solid lines with dots in the right panels). A qualitative assessment of the 3:1 artery-to-vein ratio in the vascular models can be made by observing that the arterial CBV consistently exceeds the venous CBV across cortical depths—except at the superficial lamina, where the highest CBV is accounted by the pial veins.

The local inhomogeneous frequency shifts produced by the four realistic vascular models are depicted inFigure 2for an exemplary oxygen saturation level and hematocrit (SO_2_vein = 78% and Hct = 45%). Two orthogonal planes are shown: one approximately 20 μm deep from the cortical surface (top plane) and the other around 500 μm deep relative to the cortical pial surface (middle plane). Near the cortical surface, substantial frequency field inhomogeneities arise from the macrovasculature, primarily pial veins, with minor contributions from smaller vessels. Conversely, microvessels dominate the middle planes, resulting in slight alterations to the frequency field homogeneity. Intracortical vessels (arteries and veins penetrating across the cortical depth) generate some slightly larger inhomogeneous frequency shifts compared to the ones generated by the capillary bed; however, these perturbations depend on the orientation of the vessel with respect to the main magnetic field. In general, the distinct macrovascular architecture at the pial level creates specific local frequency field signatures, while the middle planes exhibit comparable degrees of induced inhomogeneity across the models.

**Fig. 3.**
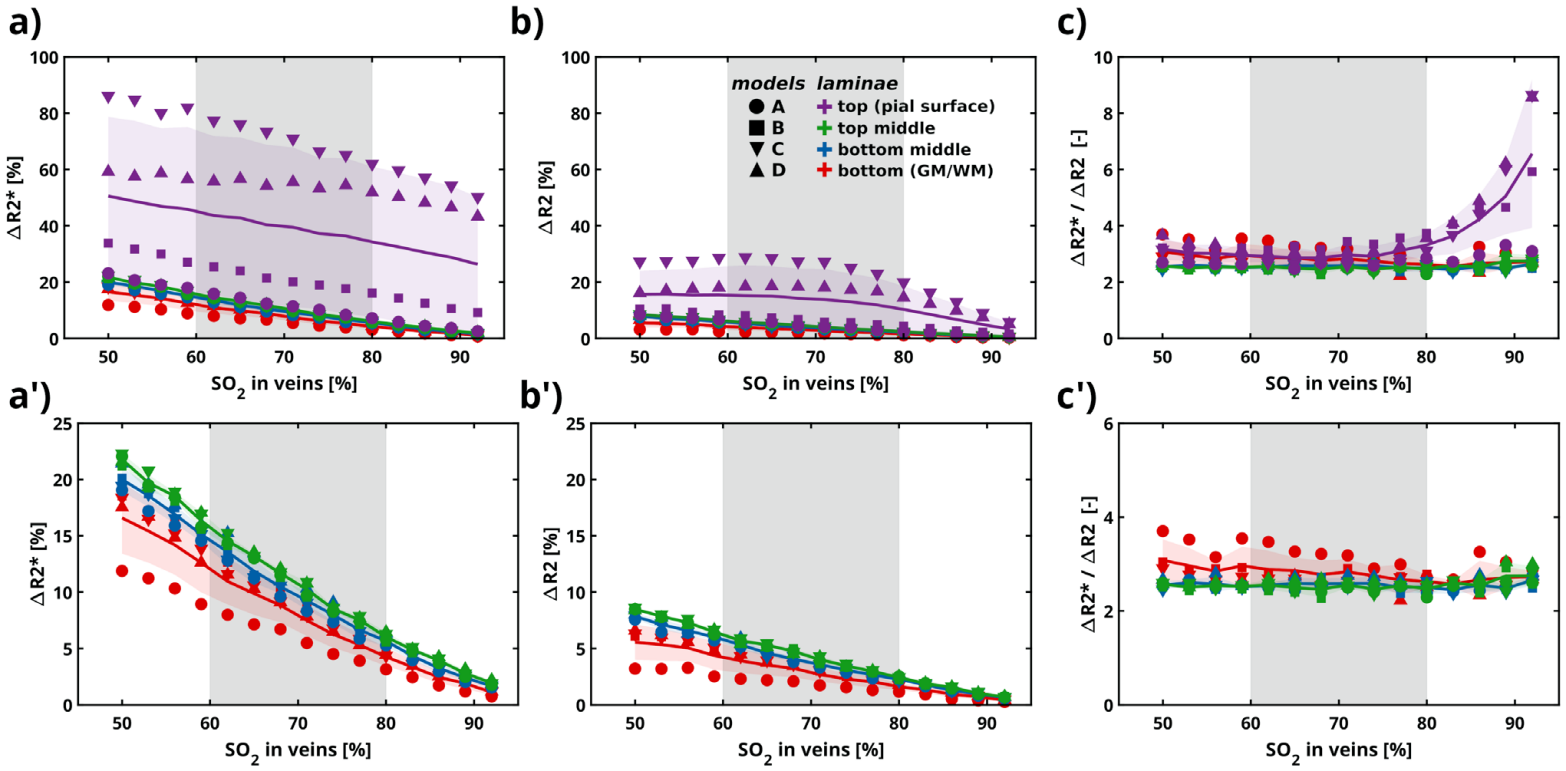
Lamina-dependent dephasing in terms of the (a) relative ΔR2* (TE = 27 ms) and (b) relative ΔR2 (TE = 50 ms) relaxation rates computed for different SO_2_values and a vessel dependent Hct (according to a systemic Hct set to 45%) at 7T. (c) Relative ΔR2* to ΔR2 ratio. (a’), (b’), and (c’) are similar to (a), (b), and (c), with the difference being the removal of the top lamina to better visualize the behavior of the remaining laminae. Each solid line represents the mean value, while the shaded area indicates the standard deviation computed through repeated Monte Carlo simulations averaged across all vascular models. The scatter points represent the average behavior per lamina for each vascular model. The grey shaded region, encompassing an SO_2_range between 60% and 80%, reflects the plausible physiological values anticipated in human fMRI data.

Figure 3illustrates the lamina-dependent dephasing, in terms of relative ΔR2* and ΔR2 relaxation rate, relative to the intrinsic relaxation rate of tissue (i.e., in the absence of any local susceptibility differences)—dependent on various SO_2_values and a vessel-dependent Hct based on a systemic Hct set to 45% (Section 2.3) for the four vascular models.

From the 16 arbitrarily defined laminae, the averages in four groups are displayed (laminae 1–4: top (comprising the pial surface); laminae 5–8: top middle; laminae 9–12: middle bottom; and laminae 13–16: bottom (comprising the GM/WM boundary)).

All vascular models consistently exhibited a linear behavior for the top-middle, middle-bottom, and bottom laminar groups in their relative ΔR2* and ΔR2 change across various SO_2_levels (seeFig. 3a’ andb’), but not for the top lamina group—the top laminar group exhibits a distinct trend that varies with oxygen saturation, where increasing SO_2_results in a nonlinear decrease as compared to the rest of the laminae. The rate of change (slope) of the relaxation rate for each laminar group, excluding the top group, obtained from linear fitting (mean value and the 95% confidence interval) is as follows: for ΔR2*, lamina top-middle has a slope of -25.12 [-24.11, -26.13], lamina middle-bottom: -18.91 [-17.57, -20.26], and lamina bottom: -13.88 [-12.24, -15.52]; for ΔR2, lamina top-middle has a slope of: -8.45 [-8.22, -8.67], lamina middle-bottom: -7.49 [-7.06, -7.92], and lamina bottom: -5.29 [-4.64, -5.94]. Both ΔR2* and ΔR2 show decreasing slopes from top-middle to bottom laminae. The change is more pronounced in ΔR2* in comparison to ΔR2, that is, ΔR2* shows larger slope differences (steeper decline). Moreover, the confidence intervals are wider for ΔR2* than for ΔR2, indicating greater variability in the slopes for ΔR2*. This implies that the measurements of ΔR2* have larger contribution from all vascular compartments and ΔR2 being more specific to certain vascular compartments.

The statistical analysis of the slopes across laminar groups for both ΔR2* and ΔR2 reveals statistically significant differences (p < 0.05) in their response to varying SO_2_levels. Pairwise comparisons show that for ΔR2* slopes, the top-middle lamina differs significantly from the middle-bottom (p ≈ 3.16 × 10^−8^) and bottom lamina (p ≈ 1.11 × 10^−12^), with the smallest p-value indicating a particularly marked difference between the top-middle and bottom layers. Similarly, the middle-bottom versus bottom laminae comparison yields a significant result (p ≈ 4.81 × 10^−5^). For ΔR2 slopes, the trend is consistent, with significant differences between top-middle and middle-bottom (p ≈ 4.31 × 10^−4^), top-middle and bottom (p ≈ 3.16 × 10^−10^), and middle-bottom and bottom (p ≈ 3.77 × 10^−6^). These low p-values indicate that each laminar group exhibits a statistically distinct relationship between SO_2_levels and relative relaxation rates, with the top-middle layer being the most sensitive to SO_2_changes, followed by a decreasing sensitivity toward the bottom layer. Therefore, we can infer that the slope is substantially different between laminar groups both within each pulse sequence (GE and SE) and between the two pulse sequences.

On the other hand, the top laminar group presents a different trend dependent on SO_2_level. Increasing SO_2_within the top lamina led to a nonlinear decrease for both ΔR2* and ΔR2, with ΔR2 showing a more pronounced decrease compared to ΔR2* (seeFig. 3a and b). Additionally, the standard deviation for ΔR2* and ΔR2 for the top lamina is larger compared to the rest of the laminae. Taking a conservative approach, we opted for a linear fit to characterize the results, the slope for the top lamina is as follows: for ΔR2*, lamina 1 (top) has a slope of -38.12 [12.93, -89.17]; for ΔR2, lamina 1 (top) has a slope of -29.19 [-6.27, -52.10].

ΔR2* was approximately 2 to 4 times larger than ΔR2 across all SO_2_levels (seeFig. 3c). This proportionality is kept for the top-middle, middle-bottom, and bottom laminar groups. The top lamina showed an increased ΔR2* as compared to ΔR2 (~4 to 8) at higher SO_2_values—in the range of 80% and above (seeFig. 3c).

InFigure 4, we illustrate the cortical depth-dependent changes in relative relaxation rates (a) ΔR2* (TE = 27 ms) and (b) ΔR2 (TE = 50 ms) across different models, highlighting the distinct properties of GE and SE techniques to cortical-layer-specific vascular properties. Each solid line in the results represents the mean value, while the shaded area represents the standard deviation computed by the Monte Carlo simulation averaged across all the SO_2_levels and an Hct set to 45%. The scatter points represent the average behavior per lamina for each vascular model.

**Fig. 4.**
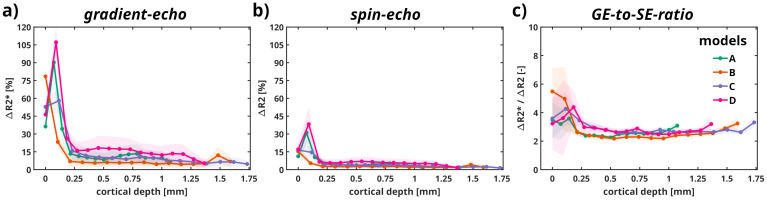
Relative changes in relaxation rate across cortical depth. (a) ΔR2* (TE = 27 ms) and (b) ΔR2 (TE = 50 ms) is depicted for all vascular models. The pial surface is positioned at 0 [mm] for all models. Panel (c) shows the resulting ΔR2*/ΔR2 ratio. Solid lines represent mean values, with shaded areas indicating the standard deviation across all SO_2_levels. Dots denote the averaged laminar contribution as individual sample points.

Both techniques exhibit a pronounced peak near the cortical surface (0 mm), where large vessel contributions are dominant, followed by a rapid decline with increasing cortical depth, reflecting a transition to microvascular contributions. GE signals (ΔR2*) in panel (a) are generally larger in magnitude compared to SE signals (ΔR2) in panel (b), consistent with the greater sensitivity of GE to all types of vessels. Differences between models become more apparent at greater depths, with model D showing relatively higher ΔR2* and ΔR2 values for both techniques. Panel (c) presents the ratio of ΔR2*/ΔR2 as a function of cortical depth, which peaks near the cortical surface and gradually stabilizes or fluctuates slightly with depth, reflecting the differential sensitivities of the two imaging techniques. This ratio highlights how GE signals are more prominent near the surface, likely due to contributions from larger pial vessels, whereas SE signals are more microvascular-sensitive.

Analysis of the deeper parts of the cortex (from 250 µm to approx. 1.75 mm), shows that the ΔR2* and ΔR2 relaxation rates follow a linear trend, with decreasing values across cortical depth. Linear fitting results in the mean and 95% confidence interval as follows: for GE: model A: -0.24 [1.35, -1.85]; model B: -0.16 [0.91, -1.25]; model C: -0.15 [0.84, -1.14]; model D: -0.19 [1.06, -1.45]; and for SE: model A: -0.08 [0.56, -0.73]; model B: -0.05 [0.37, -0.49]; model C: -0.05 [0.34, -0.45]; model D: -0.06 [0.44, -0.57].

All pairwise comparisons yield high p-values (all above 0.95), indicating no statistically significant differences between the slopes across models for both GE and SE relaxation rates. This is further supported by overlapping 95% confidence intervals for the slopes of each model pair compared. This suggests that the trends in relative relaxation rates across cortical depth are similar across models A, B, C, and D, without substantial variation in the linear slope estimates. However, there are statistically significant differences between imaging techniques (p < 0.05).

InFigure 5, we display cortical depth-dependent relative BOLD signal changes using GE and SE techniques across all models, revealing how these methods differ in sensitivity to vascular and tissue properties at varying depths.

**Fig. 5.**
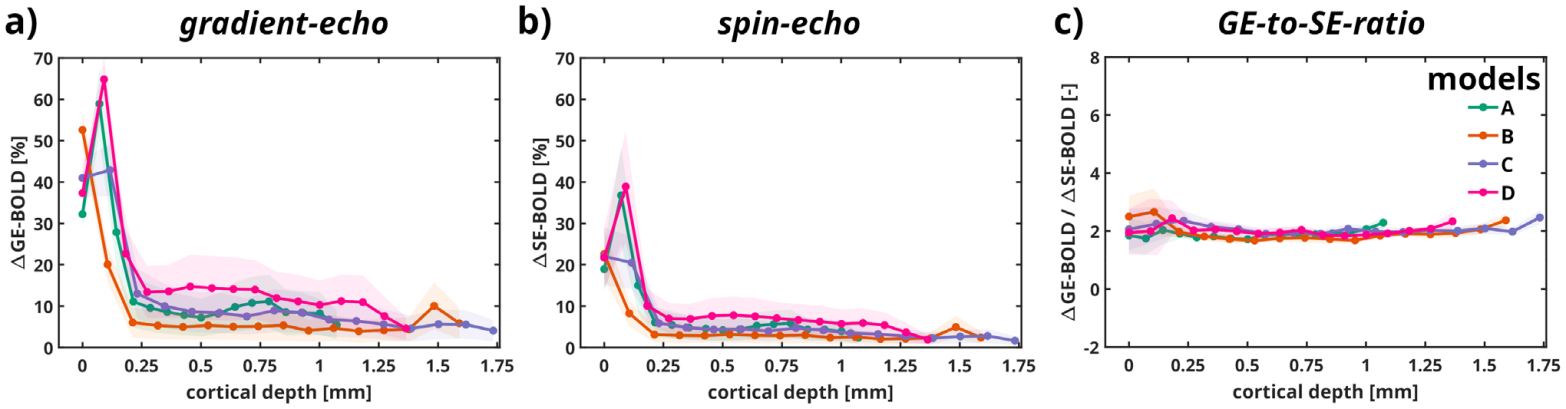
Relative BOLD signal changes for (a) GE (TE = 27 ms) and (b) SE (TE = 50 ms) across cortical depth in [mm] depicted for all vascular models. (c) Ratio of GE-BOLD and SE-BOLD. The pial surface is positioned at 0 [mm] for all models.

In panel**(a)**, the relative GE-BOLD signal peaks near the cortical surface (0 mm depth) and decreases sharply with depth, reflecting the dominance of large pial vessels at the surface and diminishing contributions from microvasculature at deeper layers. Among the models, model D shows consistently higher relative GE-BOLD signals at greater depths compared to the others, which exhibit similar profiles. Panel**(b)**illustrates the SE-BOLD signal, which also peaks near the surface but declines more gradually with depth compared to GE-BOLD, consistent with the SE sensitivity to microvascular contributions over large vessels. Similar to GE-BOLD, model D shows higher SE-BOLD signals at deeper cortical depths compared to models A, B, and C. Panel**(c)**presents the ratio of GE-BOLD to SE-BOLD across depths, which is highest near the cortical surface due to greater sensitivity of GE to superficial large vessels. This ratio stabilizes at deeper cortical depths, reflecting a convergence of GE and SE sensitivities as microvascular contributions dominate.

Similar to the statistical analysis conducted for the relative change in relaxation rates, all pairwise comparisons of the slopes across models for both GE and SE BOLD signal changes yield high p-values (all exceeding 0.95), indicating no statistically significant differences across models. However, statistically significant differences are observed between the imaging techniques (p < 0.05).

The extravascular, venous, and arterial intravascular contributions to GE and SE signals are depicted inFigure 6. Solid lines represent averaged values, and shaded areas indicate the standard deviation for all vascular models. The scatter points represent the average behavior per lamina for each vascular model. In the GE signal contribution, the dominant signal comes from the extravascular compartment (on average, ~98% signal contribution), with minimal intravascular contributions from both arterial and venous sources, each less than 2%. Nevertheless, the model D showed substantial venous signal contribution at higher SO_2_values (~3–5% signal contribution). Conversely, in the SE signal, intravascular signal contributions, particularly from venous sources, are more substantial. Depending on the SO_2_level, the contribution of the arterial component is minimal—less than 0.5%, while the venous component can reach, on average, approximately 4–6% dependent on the vascular architecture. Consequently, the SE extravascular compartment constitutes an average signal contribution ranging from 94% to 96%, depending on the SO_2_level. When observing the behavior of each lamina and model individually, the top lamina (i.e., pial surface) shows the largest changes in venous intravascular contribution for all vascular models, with model D displaying particularly large differences—up to 15% intravascular venous contribution. The intravascular arterial contribution, as expected, is slightly reduced for higher oxygen saturation values for both GE and SE.

**Fig. 6.**
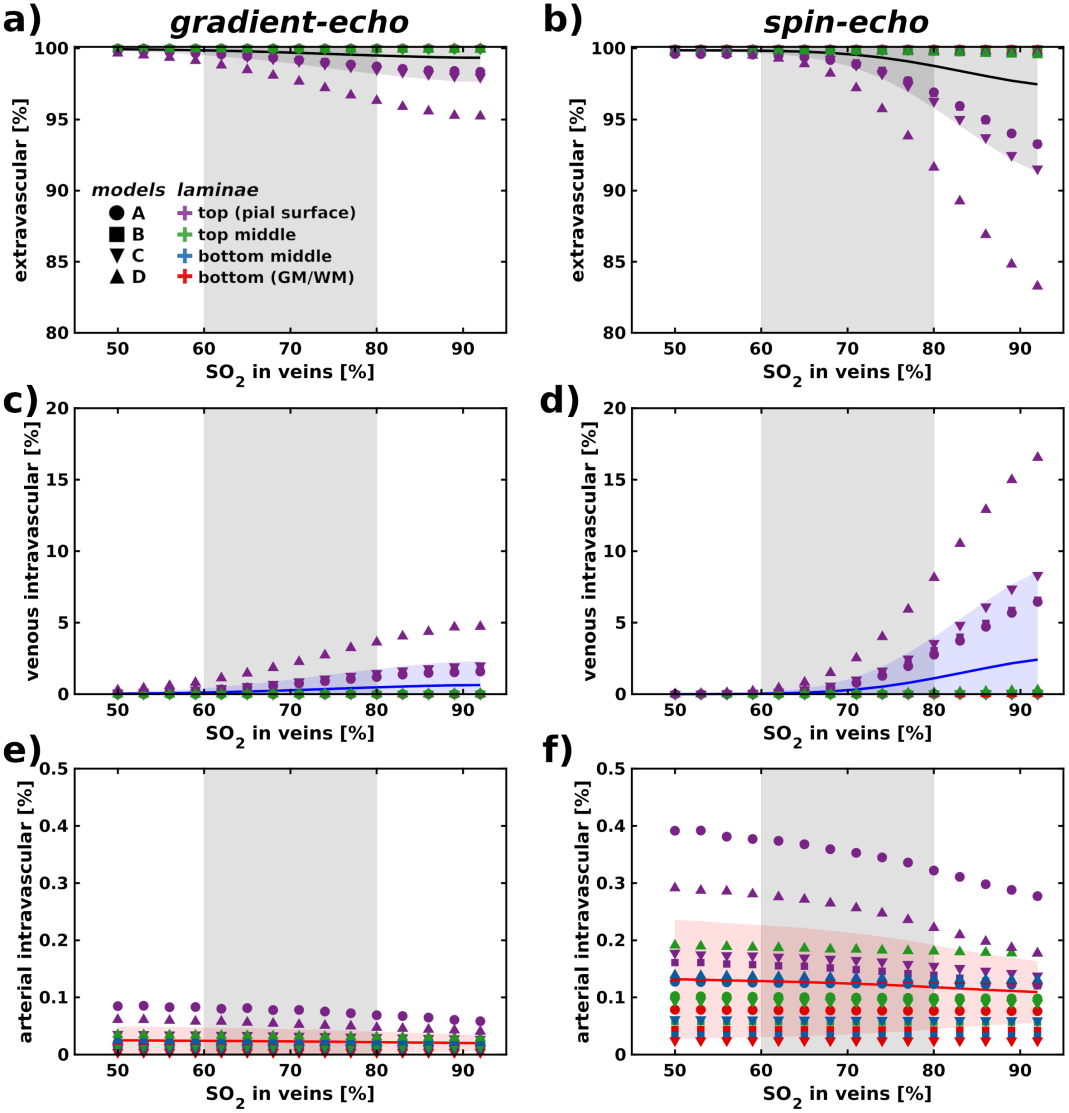
(a–b) Extravascular contribution, (c–d) venous intravascular contributions, and (e–f) arterial intravascular contribution to GE and SE signals for various SO_2_levels and a vessel-dependent Hct (systemic Hct set to 45%). Each solid line represents the mean value, while the shaded area indicates the standard deviation computed through repeated Monte Carlo simulations averaged across all vascular models. The scatter points represent the independent average lamina behavior for each vascular model. The grey shaded region, encompassing an SO_2_range between 60% and 80%, reflects the plausible physiological values anticipated in human fMRI data.

InFigure 7, we display the relative BOLD signal change dependent on Hct—relative to the computed signal at a hematocrit of 45%—across laminar groups for GE and SE for all vascular models. It is important to note that the vessel size-dependent hematocrit is driven by the simulated systemic hematocrit.

**Fig. 7.**
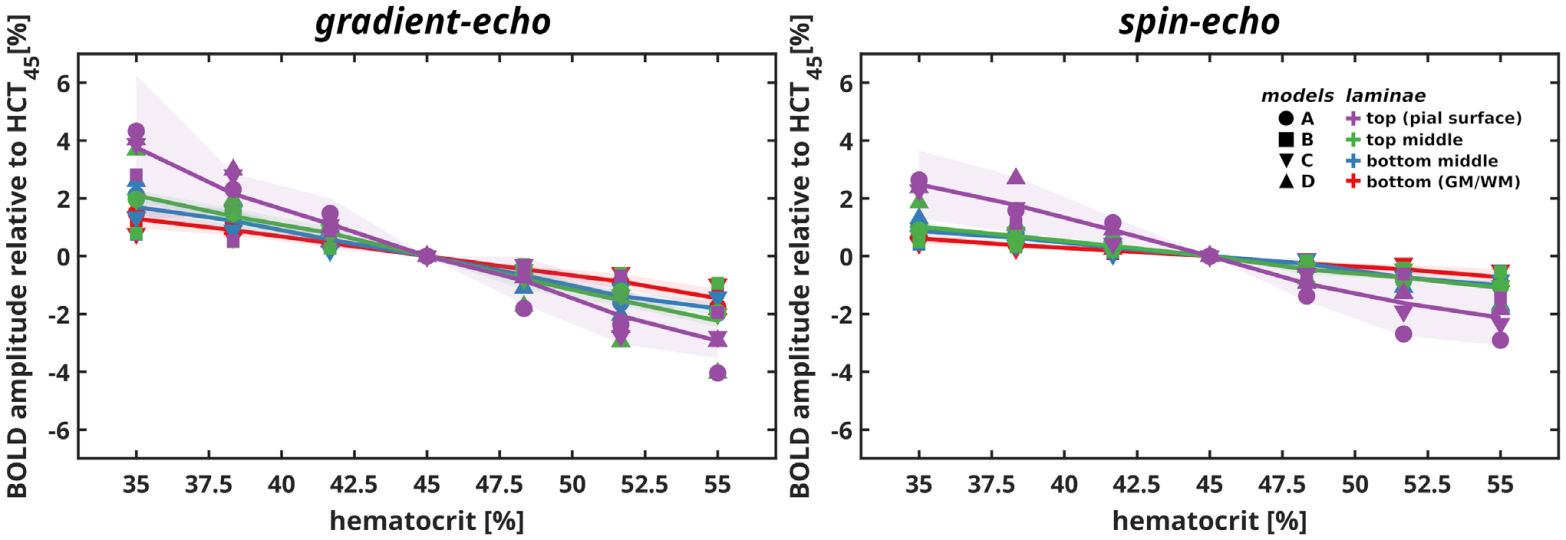
Relative BOLD signal change dependent on Hct—relative to the computed signal at a hematocrit of 45%—across cortical depth for GE and SE. Hct is vessel dependent and set according to the systemic Hct (x-axis; seeSection 2.3). Solid lines represent the mean value, while the shaded area indicates the standard deviation computed through repeated Monte Carlo simulations averaged across all vascular models. The scatter points represent the independent average lamina behavior for each vascular model.

All vascular models consistently demonstrated a relative linear trend in the BOLD signal amplitude across all lamina groups dependent on the Hct for both GE and SE readouts (seeFig. 7a and b, respectively). The higher the Hct, the smaller the BOLD signal change. This implies that higher Hct in the vasculature induces larger inhomogeneous magnetic fields and a substantial reduction of the relative change of the magnetic resonance signal.

Linear fitting results in rates of change (slope) for each lamina as follows: for GE, lamina top has a slope of -0.292 [-0.152, -0.433], lamina top-middle of -0.162 [-0.157, -0.166], lamina middle-bottom of -0.133 [-0.116, -0.150], and lamina bottom of -0.102 [-0.082, -0.121]; for SE, lamina top has a slope of -0.209 [0.096, -0.515], lamina top-middle of -0.084 [-0.077, -0.090], lamina middle-bottom of -0.075 [-0.069, -0.081], and lamina bottom of -0.053 [-0.037, -0.067]. Slopes represent the average value and the 95% confidence interval for all models. All pairwise comparisons yield significantly low p-values (all below 0.95), indicating statistically significant differences between the slopes across lamina groups.

For both GE and SE, the slope (rates of change) decreases from the top to the bottom layers, that is, the magnitude of change is the smallest at the bottom lamina compared to the top lamina. The slopes are more negative in GE compared to SE and thus the rates of change are steeper for GE across all laminae. In SE, the variance is narrower as compared to GE. This suggests that the vascular contribution to SE is relatively similar across cortical depth. On the other hand, GE presents a greater variability, indicating contributions from different vessel types.

## 4 Discussion

### 4.1 General discussion

The aim of this study is to investigate the influence of cortical vascular architecture, vessel size-dependent hematocrit, and SO_2_levels on the intravascular and extravascular signal contributions to the cortical depth-dependent GE and SE BOLD signals at 7T. To achieve this, we utilized four realistic 3D vascular models derived from two-photon microscopy imaging data obtained from mice and, by adjusting the artery-to-vein ratio, we simulated features of the human cortical vasculature. We simulated different oxygen saturation states, vessel size-dependent hematocrit levels, and biophysical interactions between blood and tissue.

A key finding is that differences in the local intra-cortical vascular architecture do not significantly (statistically) influence the shape and amplitude of BOLD profiles. For BOLD fMRI experiments, this finding implies that signal profiles within a cortical region of a given angioarchitecture (e.g., primary sensory cortex regions examined here) can be averaged within a given layer without introducing substantial errors in the results. The second main finding is that SE may contain pial vessel contributions particularly at high SO2 (>80%). This suggests that in experiments, for instance using hyperoxia, or in cases where the venous oxygen saturation is expected to be high, SE may not be as specific to the microvasculature as expected. Third, the extravascular contribution primarily dominates the BOLD signal formation in GE, with the venous intravascular contribution playing a secondary role, while the arterial intravascular contribution remains minimal. In contrast, SE exhibits a reduced extravascular contribution compared to GE, while intravascular contributions play a more significant role in the BOLD signal. Finally, we found that the BOLD signal changes increase with decreasing Hct. This finding is important for modeling studies of laminar BOLD, as assuming a single Hct value across cortical depth and different vascular compartments may not yield correct results. With respect to BOLD measurements, differences in Hct will also contribute to the BOLD amplitude across cortical depth, but this should always be considered in light of other mecahnisms such as CBF and CVB changes which may counteract the effect of Hct level on the BOLD amplitude.

The vascular architectures of the models used consist of arteries, microvessels (capillaries, small arterioles/venules), and veins. The capillary bed across the four models exhibits similar vascular properties, such as vessel radius (seeBáez-Yáñez et al., 2017;Blinder et al., 2013), relatively similar baseline CBV (seeFig. 1), and, to a certain degree, comparable topologies. It is important to note that the vascular architectures were derived from the same cortical region (the primary sensory cortex), but from different mice. The microvascular similarities suggest that, despite the limited number of vascular models, the MR signal contribution from this compartment should be comparable across simulations. This was demonstrated by the relatively similar patterns in frequency shifts at the middle plane of the voxel (seeFig. 2) and in the relative relaxation rates and BOLD signal changes for SE, particularly evident in deeper cortical laminae, for which microvessels primarily contribute to the BOLD signal formation.

The macrovascular architecture differs across the models. This resulted in inhomogeneous frequency shifts observed at the top plane of the voxel (seeFig. 2), highlighting the significant impact of large vessel topology on the MRI signal formation in a voxel. Consequently, the contribution from large vessels may not be generalizable, particularly at high spatial resolutions. It could be that if larger pial vessels are not accounted for, brain activations near the pial surface might be missed. In this situation, approaches such as excluding these vessels from the analysis (e.g.,Fracasso et al., 2018), contrasting between experiment conditions (e.g.,Kok et al., 2016), or regressing large vessel signals out using modeling approaches (e.g.,Markuerkiaga et al., 2016;Havlicek et al., 2017) can help in resolving brain activations near the pial surface. It is also possible that if signals from larger pial vessels are not accounted for in the analysis, they might create false positives. This would hold for GE as well as SE if venous SO2 is high. In sum, care must be taken in data analysis and interpretation to adequately account for purely vascular related BOLD signals originating from the larger pial vasculature.

Our findings also indicate that the relative relaxation rate for both GE and SE imaging techniques for top-middle, middle bottom, and bottom laminae exhibits a linear decrease with increasing oxygen saturation levels (seeFig. 3a and b). This effect is more pronounced in GE as compared to SE (by a factor of approximately three). The statistical diffence between deeper laminae suggests that the vascular contribution across cortical depth is influenced by the composition in vasculature, that is, differences in microvascular density have an impact on the lamina behavior.

The top laminae do not exhibit a linear behavior with increasing oxygen saturation levels. This can be attributed to the contribution of large vessels, particularly the venous compartment, in the vascular models. Moreover, at higher oxygen saturation levels (>80% SO_2_in veins), the top laminae display differing behaviors for GE and SE. Specifically, GE shows larger relative relaxation rates as compared to SE, with GE values being 4 to 8 times higher for large oxygen saturation values given the large venous blood volume contribution and venous intravascular component.

Furthermore, the relative relaxation rates across cortical depth reveal a substantial contribution from larger vessels for both GE and SE in the top laminae, that is, within the first 250 µm of the vascular models (seeFig. 4). On average, ΔR2* values (GE) are 3 to 4 times larger than ΔR2 (SE) in the pial surface. In deeper laminae (top-middle, middle-bottom, and bottom), the relative relaxation rates exhibited a linear decrease with cortical depth, with a ΔR2*/ΔR2 ratio of approximately 2 in line with experimental observations (Siero et. al. (2013)). The decrease in the ΔR2*/ΔR2 ratio in deeper cortical depth implies an increased microvascular weighting for GE at these cortical depths. Although the relative relaxation rates across cortical depth were significantly different between GE and SE, they were non-significant between the four models for each imaging technique, suggesting that the variations in vascular architecture between all models do not impact the BOLD signal profile.

The relative BOLD signal change exhibits the same linear trends in the deeper laminae, and these were also not significantly different between the four models, but significantly different between GE and SE. This indicates that the vascular topology in deeper layers did not significantly influence the shape of the BOLD profile across cortical depth for each imaging technique. On average, GE reached a peak of 8% signal change, while SE, due to its reduced specificity to macrovascular contributions, peaked at an average of 4% relative signal change (seeFig. 5). According to our results, the venous intravascular contribution to the spin-echo signal within the plausible physiological range can reach approximately 6–10%, depending on the vascular model. In contrast, the arterial intravascular components contribute minimally to the overall signal. The majority of the signal arises from the extravascular contribution, which dominates the composite BOLD signal formation. We propose that this behavior reflects a specific combination of vessel size and diffusion effects, which situates the system within the diffusion-narrowing and static-dephasing boundary regime. This regime represents a transitional state where the interplay between diffusion-driven spin dephasing and static field inhomogeneities within the vascular architecture becomes particularly significant. Such a configuration may enhance sensitivity to microvascular geometry and physiological changes as shown at the pial surface of the vascular models.

Interestingly, the SE refocusing pulse did not entirely eliminate the contribution of large vessels at the pial surface (within the cortical depth range of 0 µm and 250 µm), indicating that SE BOLD may not fully mitigate macrovascular effects near the cortical surface. This might be explained by the particular morphology and arrangement of vessels near the pial surface and the diffusion effects—a mixture of diffusion narrowing and static dephasing regime (Dumoulin, 2017;Polimeni et al., 2010).

Nevertheless, simulations using the mono-sized randomly placed and oriented cylinder models (Kiselev, 2001) show that large vessel contribution is still present in the SE BOLD signal change but reduced as compared with the GE BOLD signal change within the plausible physiological SO_2_range as shown in theSupplementary Figure S2. It is worth noting that the values cannot be directly compared between GE and SE because the SE baseline primarily reflects small vessel contributions (given the SE microvascular specificity), whereas GE includes contributions from all vascular types. Additionally, specific CBV values for the micro- and macro-vasculature vary with voxel size and position across cortical depth. Hence, based on our simulations, we determine that the specificity and signal amplitude of BOLD fMRI signals, whether obtained using GE or SE sequences, are dependent on the spatial distribution of large vessels on the pial surface, and to a lesser extent on their distribution across cortical depth.

The relative BOLD signal change in this study focuses on the extravascular and intravascular signal contribution. The extravascular component arises from the interaction between diffusing spins and magnetic field changes due to susceptibility differences in the vascular compartments and due to differential SO_2_levels. The intravenous contribution to the BOLD signal might be disregarded for GE readouts due to the relatively short T2*_0_relaxation time constant of oxygenated blood at ultra-high magnetic fields, as indicated byUludağ et al. (2009)and as demonstrated in our results. Our findings also indicate that the intravascular venous contribution in SE acquisitions is significant, particularly in conditions with relatively higher (≥80%) SO_2_levels. In these scenarios, the increased oxygen saturation affects the proportion of deoxygenated hemoglobin, thereby influencing the SE signal. This highlights that even for SE acquisitions, where extravascular contributions are typically more emphasized, intravascular signals, mostly venous signal contributions, remain impactful and should be considered in fMRI analyses, especially when interpreting BOLD signal changes related to vascular oxygenation such as in gas-challenge experimental settings.

The formation of the BOLD signal is strongly influenced by hemoglobin concentration (Gould & Linninger, 2015). Variations in hematocrit across different vascular compartments can lead to differences in BOLD signal changes. Our simulations, which accounted for vessel-size-dependent hematocrit, demonstrated that BOLD signal amplitude changes linearly with hematocrit levels: The relative BOLD signal change exhibited a decreasing linear trend with increasing hematocrit levels. Furthermore, the contribution of each lamina varied, with the top laminae exhibiting a faster increase in BOLD signal compared to deeper laminae, likely due to differences in vessel architecture. This effect was more pronounced in GE readouts than in SE readouts. These findings indicate that as red blood cell concentration increases, the BOLD signal becomes progressively stronger (Hartung et al., 2018;Levin et al., 2001). It is important to note that these results were calculated relative to an arbitrary baseline state, assuming a systemic hematocrit of 45%.

Low hematocrit generally weakens the BOLD signal by reducing magnetic susceptibility differences and the intravascular contribution. However, it can amplify the sensitivity to deoxygenation in tissues if oxygen extraction increases. These effects highlight the importance of accounting for hematocrit variability when interpreting BOLD fMRI data, particularly in conditions involving anemia or altered blood composition. The influence of Hct should be accounted for with caution however, as changes in CBF and CVB may counteract the effect of Hct level on the BOLD amplitude.

The presented results align with experimental data obtained by our group (Roefs et al., 2024;Schellekens et al., 2023). These fMRI measurements were conducted in healthy volunteers under both visual stimulation and gas-challenge conditions (hyperoxia and hypoxia-induced). Potential relative changes in BOLD signals or relaxation rates in specific diseases must consider the unique vascular properties associated with each condition. For instance, in vascular dementia, vessels exhibit a limited capacity to dilate, which impacts blood flow and other hemodynamic features.

### 4.2 Modeling considerations and limitations

Gagnon et al. (2015)were among the first to demonstrate the feasibility of conducting fMRI simulations based on realistic vascular models. A key difference between their model and the four vasculatures presented in this manuscript is the broader cortical depth coverage. While the VAN model only spans a relatively small field-of-view and specifically addressed the mice vasculature, the vascular models used in this manuscript (Blinder et al., 2013) provide a larger field-of-view, offering an alternative approach to studying laminar fMRI profiles using a more extensive model that can be modified to mimic the human cortical vasculature. Moreover, the angioarchitecture differs overall, with theBlinder et al. (2013)models exhibiting greater variability in topology. Another key difference between the results presented in this manuscript and simulations described in literature (Báez-Yáñez et al., 2017) lies in the assignment of oxygen saturation levels to vascular compartments. InBáez-Yáñez et al., (2017), the same oxygen saturation value is applied uniformly to all vessel types using the Finite Perturber method (Pathak et al., 2008). In contrast, this manuscript assigns specific oxygen saturation levels to each vessel type, resulting in a more realistic and physiologically accurate simulation model.

One of the main vascular differences between humans and mice is the artery-to-vein ratio, which has an important relation to functional signals acquired with BOLD fMRI. This significant variation has been observed and extensively reported, primarily in the work ofSchmid et al. (2019), as well as other researchers (Cassot et al., 2010;Lorthois et al., 2011;Weber et al., 2008). Furthermore, the vessel diameter values selected in this manuscript fall within the range of reported vessel radii as documented byDuvernoy et al. (1981): capillaries = 2–3 μm, arterioles = 5–37.5 μm, venules = 10–62.5 μm;Lorthois et al. (2011): capillaries = 1.9–5 μm, mean radius approx. 3.3 μm;Reina-De La Torre et al. (1998): capillaries <3.5 μm, arterioles = 10–45 μm. It is expected that based on these assumptions, the computed fMRI signals will reflect similar behavior as human measurements.

A limitation of the current study is the arbitrary labeling of large vessels. After labeling the microvasculature using a threshold of 6 µm in vessel radius, the remaining ‘large’ vessels leave limited room for modifying the artery-to-vein ratio. In this study, we applied an assumed human artery-to-vein ratio to label the macrovessels based on their connectivity and tracked their paths across cortical depth. In some instances, the imposed 3:1 artery-to-vein ratio resulted in a penetrating vessel containing segments that are labeled as artery and others as vein. While this creates a discontinuity in the given vessel’s labeling, it respects the influence of the 3:1 artery-to-vein ratio on the amplitude of the laminar profile across cortical depth. While this ratio was kept consistent across all models, the specific selection of certain vessel topological configurations may influence the behavior of the laminar profiles across cortical depth. Likewise, it is crucial to consider the disparity in cortical thickness and vessel size between mice and humans (Blinder et al., 2013;Duvernoy et al., 1981;Gagnon et al., 2015;Schmid et al., 2019), though this does not influence the simulation conclusions reported here. Vessel size, in this context, influences only the specificity of the vessel’s contribution to the BOLD signal. Nevertheless, we anticipate improving our computational model to account for this disparity. One prospective method could entail virtually generating synthetic 3D vascular networks (Báez-Yáñez et al., 2024), incorporating statistical characteristics of both macrovessels and microvessels, which span approximately ~2–3 mm in cortical depth and maintain an artery-to-vein ratio consistent with the cortical properties of a specific brain region (Duvernoy et al., 1981). This approach may also be explored to investigate differences and/or similarities of BOLD signal profiels between brain regions.

The BOLD signal is affected by alterations in local CBF, CBV, and SO_2_. In this study, we have focused solely on simulating the effects of various SO_2_states and hematocrit. However, it is important to incorporate changes in CBV in future investigations, as variations in CBV significantly influence the relaxation rates (dependent on echo-time) and, consequently, the BOLD signal change (Smirnakis et al., 2007). The results fromTian et al. (2010)provide key insights into depth-dependent vascular responses in the mouse cortex, with important implications for human fMRI studies and biophysical modeling. Their findings highlight that vascular reactivity is not uniform across cortical depth, with superficial layers exhibiting stronger vasodilation than deeper layers. This has direct consequences for interpreting hemodynamic signals in high-resolution imaging techniques.

The depth-dependent vascular responses observed inTian et al. (2010)align with findings from high-resolution fMRI studies in humans (e.g.,Fracasso et al., 2018;Huber et al., 2017;Koopmans et al., 2010;Siero et al., 2013), which demonstrate that BOLD signals are strongest in superficial cortical layers. This is likely due to a combination of greater microvascular dilation in upper layers and the influence of large draining veins near the cortical surface in humans. Similar trends are evident in our results (seeFig. 5), although our simulations account only for static oxygen saturation levels and constant CBV. Despite these similarities, several key differences must be considered. The vascular architecture in humans is more complex, with a thicker cortex (~2.5–3 mm in humans vs. ~1 mm in mice). Additionally, the artery-to-vein ratio varies by brain region, meaning depth-dependent hemodynamics may not be uniform across different cortical areas. Thus, integrating high-resolution fMRI, biophysical modeling, and optical imaging or two-photon microscopy in animal models can help validate depth-dependent vascular responses across species.

Furthermore, this encourages deeper exploration of the complex relationship between neural activity, hemodynamic response, and the unique characteristics of cortical layers. While our study introduced a more realistic vascular architectural model to assess its impact on the BOLD signal change across cortical depth, we noted the relative simplicity of our hemodynamic model. Acknowledging this limitation, we recognize the necessity of adopting a more sophisticated hemodynamic model for future advancements. Advancing our understanding of hemodynamic processes will refine simulations by incorporating factors like blood flow and blood volume dynamics, oxygen transport, and metabolic regulation, leading to a more comprehensive representation of vascular function and its influence on imaging signals (Vazquez et al., 2010).

Varying the arterial SO2, for instance decreasing oxygen content, would modulate the BOLD amplitude as more vessels with deoxygenated blood would contribute to the BOLD signal. This would affect the amplitude of laminar profile in proportion to the number of arterial vessels at a given cortical depth. But, for the purpose of this study, we chose to simulate a 95% oxygen saturation assuming it represents a worst-case scenario for healthy subjects in this particular vascular compartment. We focus on varying the venous oxygen saturation which is the vascular compartment that mainly contributes to the BOLD signal in most laminar fMRI studies. However, it would be interesting to explore the effects of different arterial oxygen saturation levels, which could be investigated through controlled gas challenges. Hyperoxia is less relevant in this context, as it only minimally increases red blood cell oxygenation. Although plasma O2 levels would rise, this does not significantly drive the overall BOLD signal. On the other hand, hypoxia presents a more interesting opportunity to study the effects of arterial blood O2 across cortical depth. It is known that intracortical arteries (~80–90% saturation;Devor et al., 2007) have slightly lower oxygen levels than the larger conduit pial arteries (95%). Further, it would be particularly interesting to simulate different oxygen saturation levels and changes in arterial cerebral blood volume (CBV), such as dilation induced by functional or neurovascular hyperemia. The combined effects of varying oxygen saturation and arterial blood volume are expected to significantly impact the BOLD signal changes across cortical depth. These effects could be explored through controlled gas challenges, such as hyperoxia during visual stimuli (Roefs et al., 2024;Schellekens et al., 2023).

It has been demonstrated that the specificity of the SE acquisition scheme has a clear dependence on the echo train length used to encode the signal using EPI readouts (Goense & Logothetis, 2006;van Horen et al., 2023). In this work, we presumed signal acquisition without any influence of the imaging gradients—the duration of spatial position encoding is neglected. Thus, different parameter selection in the EPI readouts may influence the behavior of the relaxation rates and the corresponding BOLD signal changes (Goense & Logothetis, 2006). Further, the vascular specificity of SE also depends on the assumed diffusion coefficient. The diffusion coefficient at the pial surface (top laminae) might influence the computed BOLD signal changes due to the faster diffusional motion of water molecules in the CSF compartment. Future simulations should take into account these two diffusion motion regimes (Kiselev et al., 2005;Poplawsky et al., 2019a).

In this manuscript, as in many previous studies on the impact of diffusion and different vascular compartments, we assumed that exchange interactions between intravascular and extravascular spaces were negligible to simplify the model, and reduce one degree of freedom. We believe that incorporating this degree of freedom may not have a substantial impact, as the intravascular contribution of capillaries is not expected to be as significant as that of larger vessels. Conversely, the extravascular effects of capillaries are relatively important due to the narrowing diffusion regime. Furthermore, while this assumption can be explored through biophysical modeling, we assumed that the contribution is minimal, and thus, its effect on the BOLD signal is diminished. However, it would be interesting to investigate scenarios where vascular impermeability is neglected, allowing spins to diffuse between intravascular and extravascular spaces, and examining the impact on BOLD signal formation. We plan to explore these types of simulations in future work.

The amplitude of the BOLD signal would be significantly influenced by the orientation of the vasculature with respect to the angular orientation of the main magnetic field, as supported by previous studies in human subjects (Fracasso et al., 2018;Viessmann et al., 2019) and simulations (Báez-Yáñez et al., 2023;Gagnon et al., 2015). This angular dependency is directly attributed to both pial vessels and penetrating/ascending vessels. Therefore, we envision future studies to demonstrate the laminar variability in the amplitude of the BOLD signal depending on various SO_2_levels.

### 4.3 Conclusion

This study employed four realistic 3D vascular models to investigate the influence of cortical vascular architecture, vessel-dependent oxygen saturation and hematocrit states, on the intravascular and extravascular contributions to GE and SE BOLD signals across cortical depth at 7T. The intra-cortical vascular architecture had a smaller impact on BOLD profiles as compared to the pial surface. Deeper layers exhibited a linear decrease in relaxation rates with increasing oxygen saturation, with GE showing a stronger effect. In contrast, the top lamina displayed nonlinear behavior due to large vessel contributions, with GE relaxation rates up to eight times higher than SE. Hematocrit levels significantly influenced BOLD signal amplitude and depth-dependent contributions. While GE signals were dominated by extravascular effects, SE retained notable intravascular venous contributions, particularly at high oxygen saturation (>80%). These findings emphasize the importance of vascular features, hematocrit, and biophysical interactions in shaping depth-dependent BOLD signals. Understanding how physiological variations in blood properties affect fMRI measurements is crucial for accurately interpreting laminar BOLD responses and thus improving ultra-high field fMRI methodologies.

## Supplementary Material

Supplementary Material

## Data Availability

The code and data underlying the findings of this study are available from the corresponding author upon request. Access is subject to a nonexclusive, revocable, non-transferable, and limited right to use solely for research and evaluation purposes, excluding any commercial use.
