# Supplementary material for: Impact of vascular architecture, oxygen saturation, and hematocrit on human cortical depth-dependent GE- and SE-BOLD fMRI signals: A simulation approach using realistic 3D vascular networks
Source: Imaging Neurosci (Camb). 2025 May 9;3:imag_a_00573. doi: 10.1162/imag_a_00573 (PMC12319794; doi:10.1162/imag_a_00573)
Supplement: Supplementary Material [file imag_a_00573-supp.pdf]

Supplementary Figures

To validate the computational framework, we reproduced the results reported in Boxerman et al. [1995] and Kiselev et al. [1999]. Using mono-sized randomly oriented cylinders, we computed ΔR2' effects for GE and SE at 1.5T with identical parameter values used by Kiselev et al. [1999].

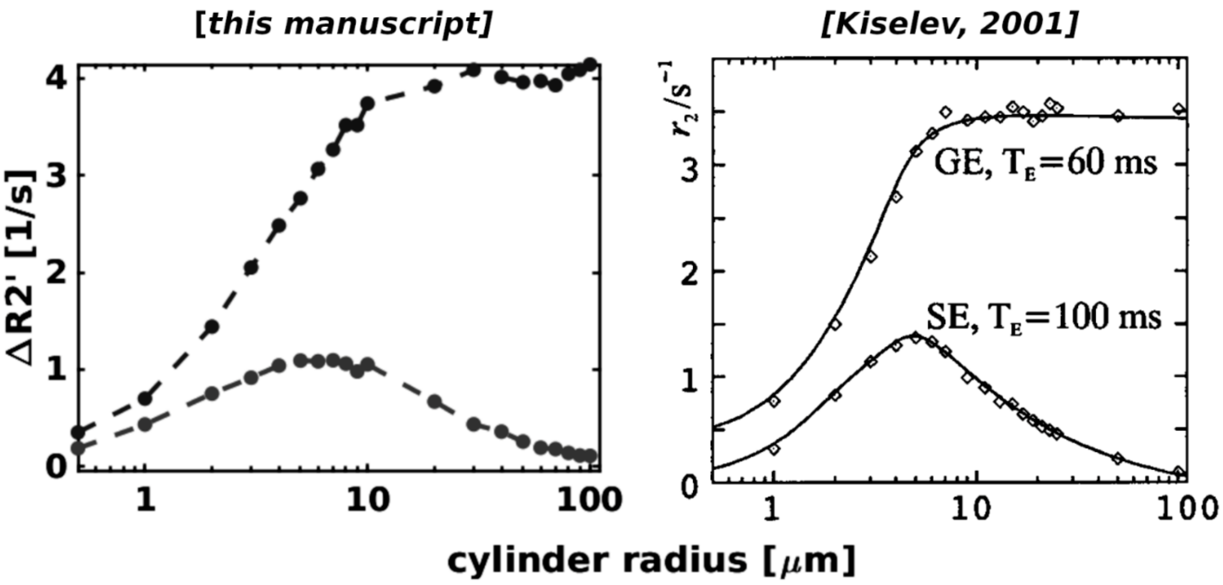

Supplementary Figure 1*. A classic result regarding vessel type contribution was reproduced. The ΔR2' decay rate (left panel) was computed to validate our Monte Carlo simulation pipeline, as presented in [Kiselev, 2001] (right panel). This ΔR2' reproduction assumes diffusion effects and an imposed susceptibility difference (Δχ = 1E-7). This simulation was performed for both GE (TE = 60 ms) and SE (TE = 100 ms) at 1.5T.*

There are differences between the Kiselev plot and our results—though minimal, within a range of 0.5 [1/s]. This slight variability is expected, as the Monte Carlo method is sensitive to the number of simulations performed and the averaging of models. Despite this, the overall behavior of our computational framework closely resembles the well-characterized results from Kiselev's work and others. This agreement validates the robustness of our approach, demonstrating that the framework can reliably reproduce established patterns while accommodating minor variability inherent to stochastic methods like Monte Carlo simulations.

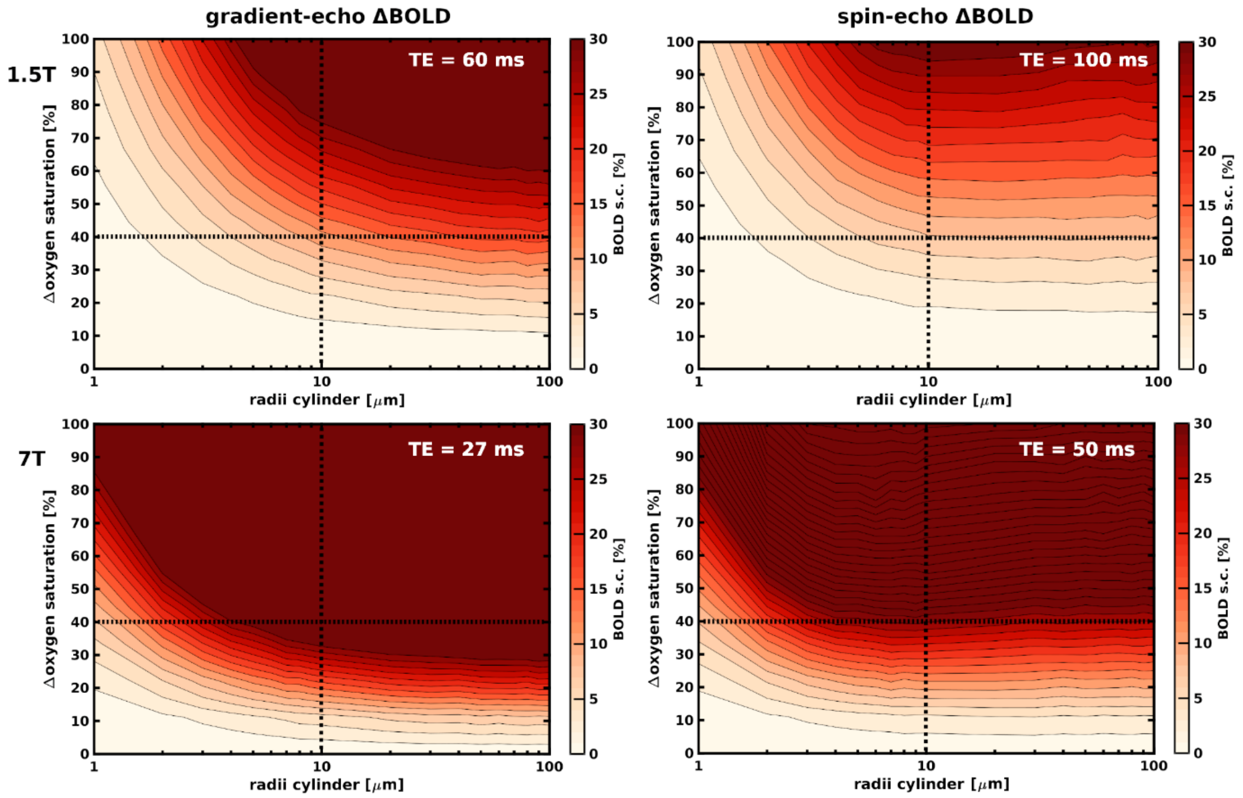

Supplementary Figure 2*. Simulated BOLD signal changes using randomly oriented monosize cylinder voxel models for GE and SE at 1.5T (top row) and 7T (bottom row) using their respective echo times as indicated on the graphs. The vertical black dotted lines represent the assumed separation between the macrovascular (large radii values) and microvasculature (small radii values) contributions to the BOLD signal change. Furthermore, we assumed that the values below the horizontal black dotted lines represent physiologically plausible values capable of generating these BOLD signal changes. Assuming the baseline state as fully oxygen-saturated (1.0), the selected value of 40% oxygen saturation corresponds to an oxygen saturation of 0.60. The parameters of the simulation are similar as described in [Kiselev et al., 2001] –D = 1E-9 m^2/s, volume fraction = 3%, HcT = 40%, time-step = 50 μs.*

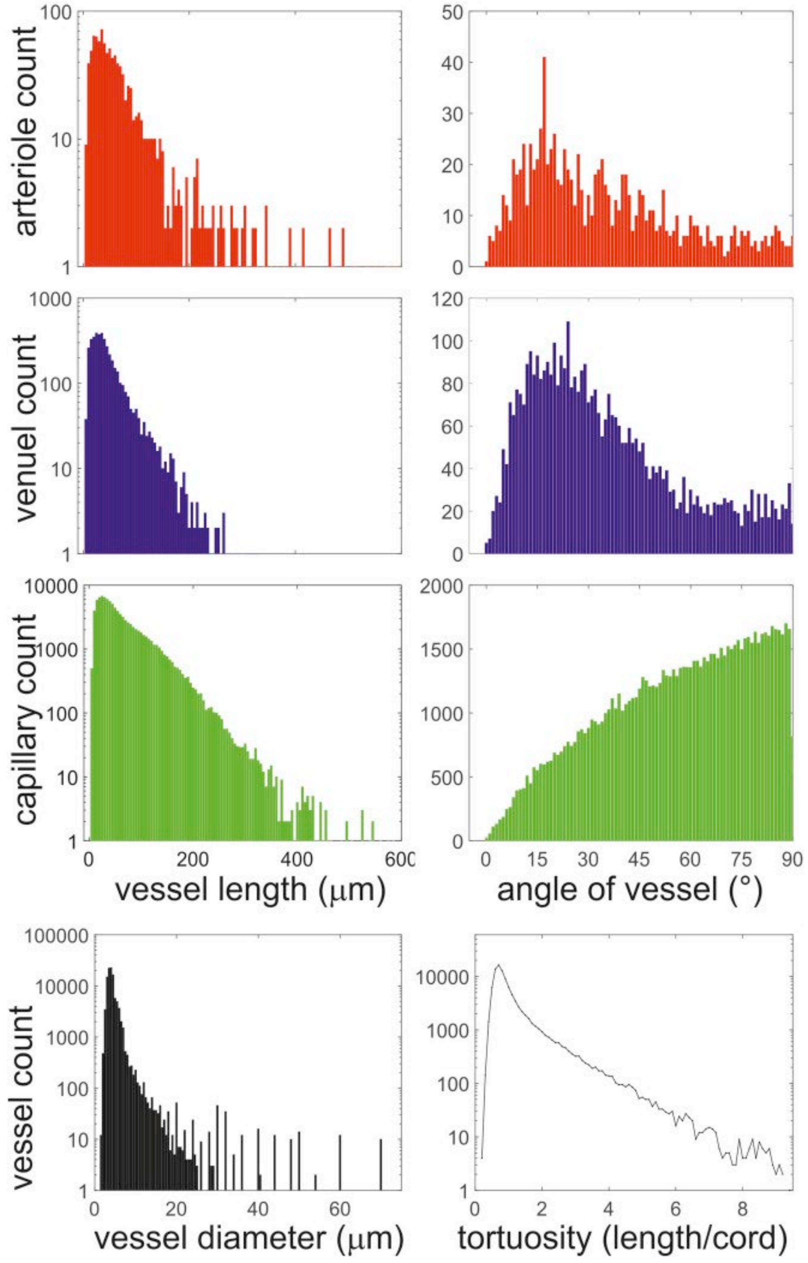

Supplementary Figure 3*. Vascular features of the four used models. Additional vessel characteristics, including diameter, length, curvature, and preferential orientation, are detailed in Báez-Yáñez et al. (2017). For clarity, we have included this figure here, as it effectively summarizes the key characteristics of all vascular models. In this manuscript, we have adjusted the artery-to-vein ratio to simulate human vasculature. As a result, the arteriole and venule counts have changed proportionally. However, since the fundamental vascular properties remain unchanged, reporting these specific count adjustments is not necessary.*
